# MPEG1/Perforin-2 Haploinsufficiency Associated Polymicrobial Skin Infections and Considerations for Interferon-γ Therapy

**DOI:** 10.3389/fimmu.2020.601584

**Published:** 2020-11-03

**Authors:** Leidy C. Merselis, Shirley Y. Jiang, Stanley F. Nelson, Hane Lee, Kavitha K. Prabaker, Jennifer L. Baker, George P. Munson, Manish J. Butte

**Affiliations:** ^1^ University of Miami Miller School of Medicine, Department of Microbiology and Immunology, Miami, FL, United States; ^2^ Division of Immunology, Allergy, and Rheumatology, Department of Pediatrics, University of California Los Angeles, Los Angeles, CA, United States; ^3^ Department of Human Genetics, University of California Los Angeles, Los Angeles CA, United States; ^4^ Department of Pathology and Laboratory Medicine, University of California Los Angeles, Los Angeles, CA, United States; ^5^ California Center for Rare Diseases, Institute for Precision Health, University of California Los Angeles, Los Angeles, CA, United States; ^6^ Division of Infectious Diseases, Department of Medicine, University of California Los Angeles, Los Angeles, CA, United States; ^7^ Department of Surgery, University of California Los Angeles, Los Angeles, CA, United States; ^8^ Department of Microbiology, Immunology, and Molecular Genetics, University of California Los Angeles, Los Angeles, CA, United States

**Keywords:** MPEG1 p.Tyr430*, perforin-2, primary immunodeficiency, membrane attack complex, interferon gamma, case report

## Abstract

**Introduction:**

Macrophage expressed gene 1 (*MPEG1*) is highly expressed in macrophages and other phagocytes. The gene encodes a bactericidal pore-forming protein, dubbed Perforin-2. Structural-, animal-, and cell-based studies have established that perforin-2 facilitates the destruction of phagocytosed microbes upon its activation within acidic phagosomes. Relative to wild-type controls, *Mpeg1* knockout mice suffer significantly higher mortality rates when challenged with gram-negative or -positive pathogens. Only four variants of *MPEG1* have been functionally characterized, each in association with pulmonary infections. Here we report a new *MPEG1* non-sense variant in a patient with the a newly described association with persistent polymicrobial infections of the skin and soft tissue.

**Case Description:**

A young adult female patient was evaluated for recurrent abscesses and cellulitis of the breast and demonstrated a heterozygous, rare variant in *MPEG1* p.Tyr430*. Multiple courses of broad-spectrum antimicrobials and surgical incision and drainage failed to resolve the infection. Functional studies revealed that the truncation variant resulted in significantly reduced capacity of the patient’s phagocytes to kill intracellular bacteria. Patient-derived macrophages responded to interferon gamma (IFN-γ) by significantly increasing the expression of *MPEG1*. IFN-γ treatment supported perforin-2 dependent bactericidal activity and wound healing.

**Conclusions:**

This case expands the phenotype of *MPEG1* deficiency to include severe skin and soft tissue infection. We showed that haploinsufficiency of perforin-2 reduced the bactericidal capacity of human phagocytes. Interferon-gamma therapy increases expression of perforin-2, which may compensate for such variants. Thus, treatment with IFN-γ could help prevent infections.

## Introduction

*MPEG1* expression is constitutive in phagocytes and inducible in parenchymal cell lines by interferons or bacterial infection (1, 2). Its function in innate immunity is vital for intracellular pathogen elimination such that *Mpeg1* knockout mice exhibit increased susceptibility to infections (2–5). *MPEG1* variants can confer an immunodeficiency hallmarked by polymicrobial infections (1).

Until now, *MPEG1* mutations have been primarily studied *in vivo* in non-human subjects, with the exception of a study by McCormack *et al*. that describes the first four clinical cases of heterozygous *MPEG1* mutations in human disease, which notably involved pulmonary infections with a preponderance of non-tuberculous *Mycobacteria* (1). Skin and soft tissue infections were not described. Here we expand the phenotype of *MPEG1* haploinsufficiency by describing a patient with polymicrobial skin and soft tissue infections bearing a heterozygous nonsense variant of *MPEG1*. We offer considerations for treating this condition with interferon gamma (IFN-γ).

### Case Description

The patient reported is a 23-year-old adopted woman with a limited history of early childhood. She has a complex medical history including bilateral sensorineural hearing loss and non-anatomic gastroparesis requiring feeds by jejunal tube. She also carries the heterozygous, pathogenic Factor V “Leiden” variant discovered after suffering multiple deep vein thromboses and pulmonary emboli, now on lifelong anticoagulation. The patient experienced numerous infections throughout adolescence and young adulthood including repeated facial impetigo, recurrent tonsillitis requiring tonsillectomy and adenoidectomy, skin abscesses, recurrent central line-associated blood stream infections (*Enterobacter cloacae*), and infections of the central line site (*Staphylococcus aureus*). Because of a concern for a primary immunodeficiency, she had been treated empirically with immune globulin, without evidence of hypogammaglobulinemia or specific antibody deficiency. At age 22, she presented with a breast abscess and cellulitis requiring incision and drainage (I&D). Over the next several months, she suffered recurrent abscesses despite serial debridement and intravenous antibiotics. During a prolonged inpatient stay for treatment of recurrent breast abscess at her local hospital, she was transferred to our institution for further evaluation and treatment. Her initial diagnostic work-up included laboratory studies that demonstrated a normal leukocyte count, mild anemia, overall normal lymphocyte counts, and a mild thrombocytosis, likely reactive to an acute infection (**Table 1**). An ultrasound of the right breast found complex fluid collections, of which the largest region measured 16 x 5 x 14 mm. A biopsy of the tissue supported a picture of acute on chronic inflammation without atypical hyperplasia or malignancy. Cultures obtained at different time points during her clinical course grew numerous organisms including *Staphylococcus pseudintermedius, Enterobacter cloacae, Klebsiella pneumoniae, Serratia marcescens, Veillonella parvula*, and *Candida* spp. (*glabrata, albicans*, and *lusitaniae*). Despite completing an extended course of appropriate antimicrobial coverage along with multiple I&D procedures, the infections did not resolve.

**Table 1:** Laboratory Results.

**Complete blood count (CBC)**	**Result**	**Units**	**Reference range**
WBC	8.00	1,000/µl	4.16–9.95
Hemoglobin	11.3	Gram/dl	11.6–15.2
Platelet	459	1,000/µl	143–398
Neutrophil	56.8	%	
Lymphocyte	33.3	%	
Monocyte	7.5	%	
Eosinophil	1.6	%	
Basophil	0.5	%	
**Inflammatory markers**	**Result**	**Units**	**Reference range**
C-reactive protein	0.3	mg/dl	<0.8
Erythrocyte sedimentation rate	16	mm/h	≤25
**Immunoglobulins**	**Result**	**Units**	**Reference range**
IgA, serum	170	mg/dl	87–426
IgG, serum	1,200	mg/dl	726–1521
IgM, serum	144	mg/dl	44–277
**Lymphocyte counts**	**Cell type**	**Result (/μl)**	**Reference range(/μl)**
CD3+	T cells	1,634	803–2,990
CD4+	Helper T cells	725	441–2,156
CD8+	Cytotoxic T cells	718	125–1,312
CD19+	B cells	441	107–698
CD56+/CD16+	NK cells	58	95–640
CD19+CD27+	Memory B cells	26.5	18–145
CD19+CD27+IgM+IgD-	IgM memory B cells	0	0–12
CD19+CD27+IgM+IgD+	Unswitched memory B cells	13.2	4–85
CD19+CD27+IgM-IgD-	Switched memory B cells	13.2	7–61
CD19+CD21low	Immature B cells	8.8	0.3–22
**Other**	**Interpretation**		
HIV-1/2 Ag/Ab screen fourth generation	Nonreactive		

## Diagnostic Assessment

**Figure 1:**
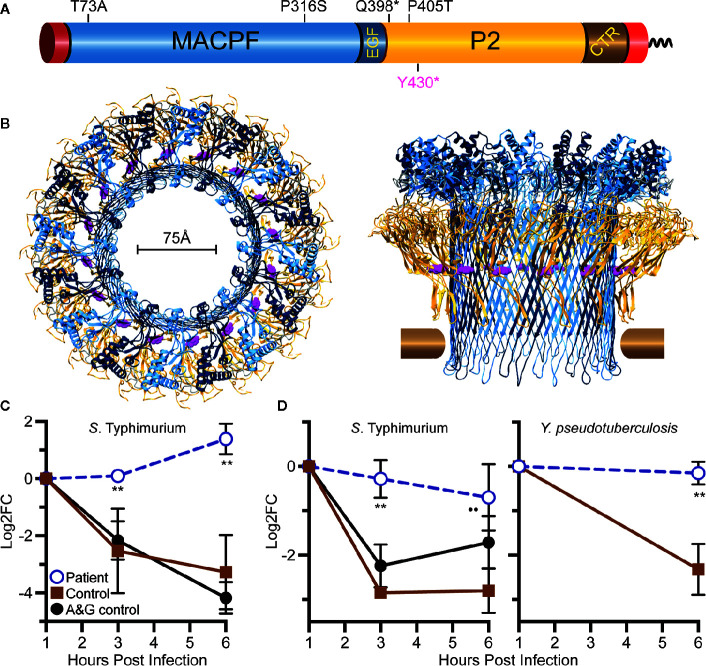
Relative location of Tyr430 within perforin-2 and demonstration of reduced killing capacity of the patient’s phagocytes. **(A)** Domain organization of perforin-2 with its signal peptide, membrane-attack-complex-perforin (MACPF) domain (blue), EGF-like domain (dark blue), perforin-2 domain (yellow), and carboxy-terminal transmembrane domain (brown, red). **(B)** Top and side view of the acid-dependent perforin-2 pore (6). Each polymer comprises 16 subunits with MACPF and P2 domains lining the interior and exterior of the polymer. Tyr430 is depicted as magenta spheres within the P2 domain. Horizontal bars represent the approximate location of the target lipid bilayer. **(C)** Neutrophil killing assay showing fold change over time of intracellular bacterial colony forming units. **(D)** Macrophage killing assay showing fold change over time of intracellular bacterial colony forming units. log_2_FC = log_2_(CFU at time X) – log_2_(CFU at time initial). **P < 0.04 to age and gender (A&G) shipping control, and *P* < 0.02 to non-matched, unrelated control.

A polymicrobial breast infection in a young woman persisting over several months despite treatment with multiple antimicrobials was suspicious for primary immunodeficiency. Flow cytometric analysis of peripheral blood lymphocytes showed mild NK cell lymphopenia, which was normal in prior and subsequent tests. Whole genome sequencing revealed a few, rare genetic variants including a nonsense variant in *MPEG1* NM_001039396.1:c.1290C>A, resulting in p.Tyr430* (dbSNP rs773347395) (**Figure 1A**), which codes for the perforin-2 protein. The CADD phred score of this variant was 36. This variant is rare, occurring in only 1 of 124,579 individuals in the Genome Aggregation Database (gnomAD) and not in homozygous form (7). A heterozygous p.D113N variant of *IKBKG* was identified as well, but because of its commonness (found in ~3% of alleles of Europeans in gnomAD), this variant was felt unlikely to be the cause of her immunodeficiency.

Given the known bactericidal function of perforin-2 and the location of this variant in the P2 domain and eliminating the C-terminal transmembrane domain, the *MPEG1* variant was suspected to be pathogenic (**Figure 1B**). If the Tyr430* truncated protein product were stably expressed it would likely be secreted rather than delivered to endophagosomes as its transmembrane domain is essential for the intracellular retention of perforin-2 (6, 8). Immunologic assays demonstrated normal neutrophil chemotaxis and extracellular bactericidal activity against *Staphylococcus aureus* (**Table 2**). However, the latter laboratory assay did not differentiate between extracellular and intracellular killing. Thus, it may not be sensitive enough to detect reduced Perforin-2 activity within phagosomes.

**Table 2:** Clinical Neutrophil Assays.

**Neutrophil chemotaxis assay before → after exposure to zymosan-activated serum**		
**Sample**	**Migration (μm)**	**Reference range**
Control	52 → 109	24–54 μm (before) → 68–114 μm (after)
Patient	52 → 100	
**Bactericidal assay (*Staphylococcus aureus*, killing at 120 min)**		
**Sample**	**Percent dead (%)**	**Reference range (%)**
Control	87	>71
Patient	86	>71

To assess the impact of the *MPEG1* variant, the subject’s neutrophils and macrophages were tested for intracellular killing. These cells were purified from anticoagulated whole blood and allowed to phagocytose *Salmonella enterica* serovar Typhimurium (*S.* Typhimurium) or *Yersinia* *pseudotuberculosis*. The phagocytes were then treated with membrane impermeable gentamicin to eliminate the extracellular population of bacteria prior to enumeration of intracellular bacteria. This assay, commonly referred to as a gentamicin protection assay, revealed that the patient’s neutrophils were unable to suppress the intracellular replication of *S.* Typhimurium (**Figure 1C**). As expected, cells from healthy controls were more effective at eliminating the intracellular pathogens. This defect was also observed with patient-derived macrophages infected with *S.* Typhimurium or *Y. pseudotuberculosis* (**Figure 1D**).

**Figure 2:**
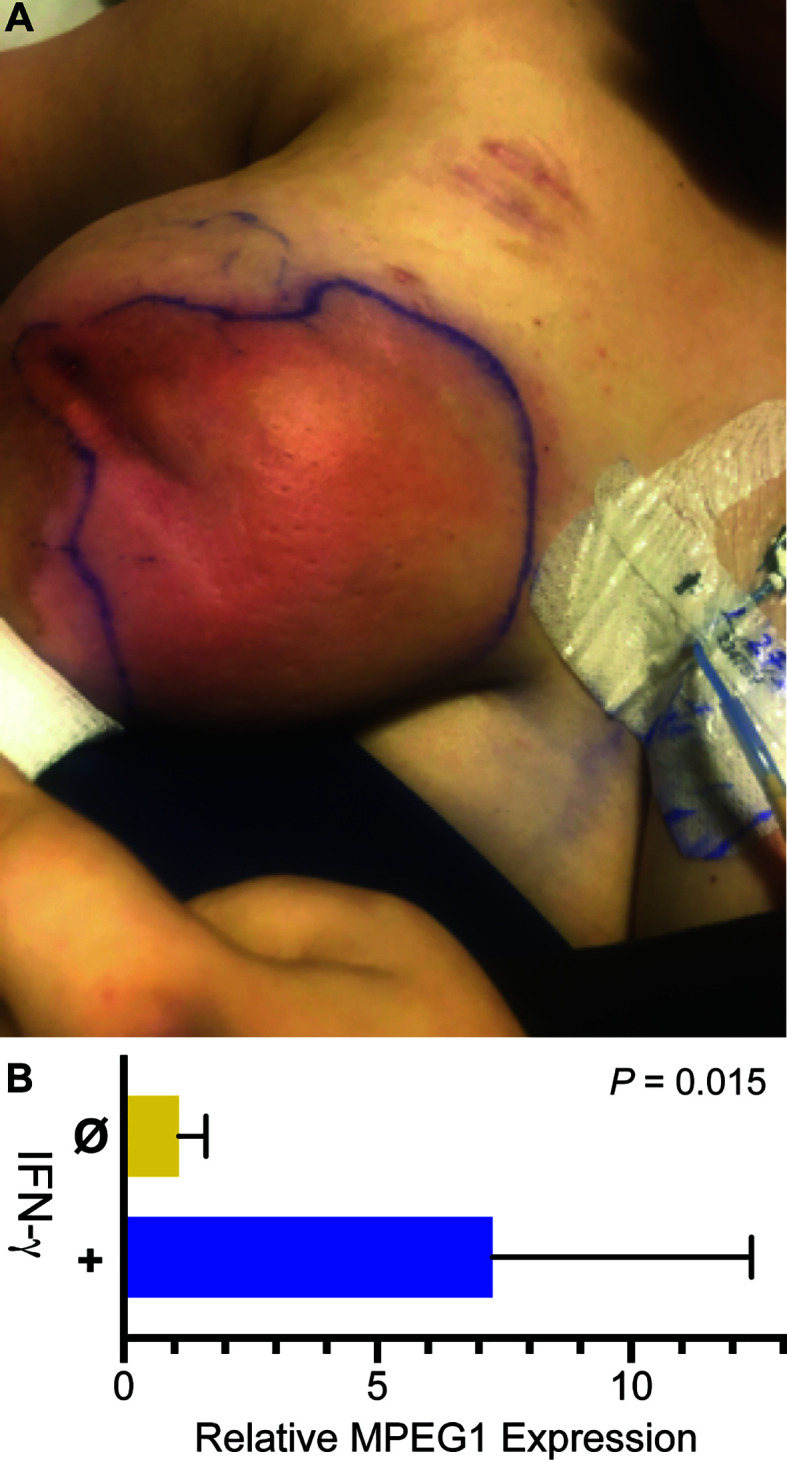
Infection and IFN-γ induction of *MPEG1*. **(A)** Purulent abscesses in the right breast. **(B)** *MPEG1* transcripts are induced after treatment *in vitro* with IFN-γ.

During the patient’s admission, she was again treated with broad spectrum antimicrobials and multiple I&D’s, however her hospital course was complicated by incomplete resolution and development of new abscesses in the right breast. Physical exam revealed multiple, tender, deep painful regions around the breast with corresponding regions of erythema (**Figure 2A**). Breast cultures during this admission again grew organisms previously present including *K. pneumoniae, S. marcescens*, and *C. glabrata*, as well as *Enterococcus faecalis* and Lactobacillus species. The patient became frustrated with months of inpatient treatments. Due to prolonged and inadequate resolution of the abscess despite multiple antibiotics and antifungals, she underwent surgical placement of antibiotic- and antifungal-impregnated calcium sulfate beads (9) and treatment with three injections of intralesional GM-CSF to promote wound healing and phagocytosis (10).

Adjuvant therapy with IFN-γ was considered next. To determine if the patient’s phagocytes were responsive to interferons, macrophages differentiated from the patient’s blood monocytes were treated with IFN-γ. Relative to untreated control, IFN-γ increased *MPEG1* expression by eight-fold (**Figure 2B**). Such upregulation may compensate for *MPEG1* haploinsufficiency by increasing perforin-2 expression from the normal allele. Facing steady worsening of the patient’s condition, we treated her with IFN-γ at a dose of 50 μg/m^2^ three times weekly for 3 weeks. The patient experienced initial improvement of symptoms with the adjuvant therapy, however she also experienced malaise, body aches, and low-grade fevers known to accompany IFN-γ therapy. Due to chronic pain and redeveloping abscesses, the patient underwent a unilateral total mastectomy using tumescent and scissor dissection, given the contraindication of cautery in the setting of her cochlear implants. Surgical pathology found inflamed granulation tissue with multiple micro-abscesses and periductal inflammation. Post-operatively she had clinical cure of infection and complete wound healing. IFN-γ therapy was continued in the outpatient setting as prophylaxis, but there have been difficulties tolerating side effects, including fever. There were no further infections in the 3 months following mastectomy.

## Discussion

This case illustrates the innate immune dysfunction in a young woman carrying a *MPEG1* genomic loss-of-function variant, presenting as difficult-to-manage skin and soft tissue infections. Perforin-2 is a member of the membrane attack complex/perforin (MACPF) family, which includes the terminal components of complement (C9) responsible for pore-forming membrane disruption to eradicate microbial invaders (6, 8). Expression of perforin-2 is upregulated by IFNs in primary fibroblasts and epithelial cell lines (11). Although the precise details are under investigation, perforin-2 is trafficked to endo/phagosomes where it polymerizes into a pre-pore structure that subsequently transitions to a pore conformation upon phagosome acidification (2, 4, 8). These pores perforate the envelope of phagocytosed pathogens, rendering them more susceptible to antimicrobial effector such as proteases, other hydrolases, and reactive oxygen and nitrogen species (2, 5). Studies of *Mpeg1* knockout mice have shown that deficiencies in this protein lead to uncontrolled, disseminated infection from the gut and skin, as well as a host of other immunologic consequences (2–4). Pathogenic variants have been reported in patients with both intracellular and extracellular pulmonary infections with *Mycobacterium, Pseudomonas, Achromobacter, Bordetella, Pneumocystis*, and *Aspergillus* species (1), suggesting that this immunodeficiency extends beyond control of intracellular organisms. An important feature of this patient’s disease is the location of the infection: the skin. Studies *in vitro* in human skin found that perforin-2 is induced during wound healing in the skin and is necessary for intracellular killing of Staphylococcus (12).

IFN-γ is a naturally occurring, pleotropic cytokine central to type-1 immunity and, as a medication, is approved for the prophylaxis of infections in the context of chronic granulomatous disease. *In vitro* studies have shown that IFN-γ induces *MPEG1* expression in an array of murine and human cell lines as well as primary cells (2, 11). Consistent with that work we found that IFN-γ significantly increased expression of *MPEG1* in patient-derived macrophages. Despite some improvement in clinical status, the widespread infection required mastectomy for resolution. The benefits of IFN-γ could thus not be fully determined. Further investigation will be required to assess if treatment with IFN-γ promotes perforin-2-mediated bactericidal activity and reduces infections in *MPEG1* deficiency.

## Conclusions

Perforin-2 plays important roles in the ability of innate immune cells to kill intracellular pathogens. We present a single case demonstrating haploinsufficiency of perforin-2 with severe, bacterial, and fungal soft tissue infections. IFN-γ therapy may be used as a potential prophylaxis for inducing the expression of the functional allele.

## Materials and Methods

### Structural Model

The structural model of perforin-2 comes from RCSB entry 6SB5.

### Killing Assays

For neutrophil killing, polymorphonuclear neutrophils (PMNs) were isolated from whole blood by Ficoll-Paque centrifugation then seeded onto tissue culture plates for 2 h. Adherent cells were subsequently infected for 45 min with *S*. Typhimurium (strain SL1344); the multiplicity of infection (MOI) was 50. Extracellular bacteria were eliminated with 50 μg/ml gentamicin. PMNs were lysed with 0.1% Triton X-100 at the specified times. Released bacteria were serially diluted for enumeration of colony forming units (CFUs) on LB agar plates supplemented with 50 μg/ml of the appropriate antibiotic.

For macrophage killing, PBMCs isolated from whole blood by Ficoll-Paque centrifugation were seeded onto tissue culture treated 100 mm petri plates in IMDM + 10% FBS media supplemented with 50 ng/ml rh-MCSF (BioLegend cat #570206). Monocytes were allowed to adhere and differentiate over 7 days with media changes every 2 days. Following differentiation macrophages were infected with the indicated pathogens; MOI 50. Gentamicin protection assays were conducted as above.

### Transcript Comparison

1x10^6^ differentiated patient macrophages were stimulated for 14 h with 100 ng/ml of human recombinant IFN-γ and subsequently lysed in RLT buffer + 1% BME. Whole culture RNA was extracted with RNeasy columns and converted to cDNA using QuantiTec Reverse Transcription Kit. Samples were run in triplicate using TaqMan probes specific for human MPEG1. Results were normalized to expression of GAPDH and presented relative to untreated macrophages. *P* value as determined by unpaired *t* test.

## Data Availability Statement

Data for **Figures 1C, D** and **2B** have been deposited in FigShare; doi.org/10.6084/m9.figshare.13122821. Further inquiries can be directed to the corresponding author.

## Ethics Statement

The studies involving human participants were reviewed and approved by UCLA IRB. The patients/participants provided their written informed consent to participate in this study. Written informed consent was obtained from the individual(s) for the publication of any potentially identifiable images or data included in this article.

## Undiagnosed Diseases Network

Maria T. Acosta; Margaret Adam; David R. Adams; Pankaj B. Agrawal; Mercedes E. Alejandro; Justin Alvey; Laura Amendola; Ashley Andrews; Euan A. Ashley; Mahshid S. Azamian; Carlos A. Bacino; Guney Bademci; Eva Baker; Ashok Balasubramanyam; Dustin Baldridge; Jim Bale; Michael Bamshad; Deborah Barbouth; Pinar Bayrak-Toydemir; Anita Beck; Alan H. Beggs; Edward Behrens; Gill Bejerano; Jimmy Bennet; Beverly Berg-Rood; Jonathan A. Bernstein; Gerard T. Berry; Anna Bican; Stephanie Bivona; Elizabeth Blue; John Bohnsack; Carsten Bonnenmann; Devon Bonner; Lorenzo Botto; Brenna Boyd; Lauren C. Briere; Elly Brokamp; Gabrielle Brown; Elizabeth A. Burke; Lindsay C. Burrage; Manish J. Butte; Peter Byers; William E. Byrd; John Carey; Olveen Carrasquillo; Ta Chen Peter Chang; Sirisak Chanprasert; Hsiao-Tuan Chao; Gary D. Clark; Terra R. Coakley; Laurel A. Cobban; Joy D. Cogan; Matthew Coggins; F. Sessions Cole; Heather A. Colley; Cynthia M. Cooper; Heidi Cope; William J. Craigen; Andrew B. Crouse; Michael Cunningham; Precilla D'Souza; Hongzheng Dai; Surendra Dasari; Joie Davis; Jyoti G. Dayal; Matthew Deardorff; Esteban C. Dell'Angelica; Shweta U. Dhar; Katrina Dipple; Daniel Doherty; Naghmeh Dorrani; Argenia L. Doss; Emilie D. Douine; David D. Draper; Laura Duncan; Dawn Earl; David J. Eckstein; Lisa T. Emrick; Christine M. Eng; Cecilia Esteves; Marni Falk; Liliana Fernandez; Carlos Ferreira; Elizabeth L. Fieg; Laurie C. Findley; Paul G. Fisher; Brent L. Fogel; Irman Forghani; Laure Fresard; William A. Gahl; Ian Glass; Bernadette Gochuico; Rena A. Godfrey; Katie Golden-Grant; Alica M. Goldman; Madison P. Goldrich; David B. Goldstein; Alana Grajewski; Catherine A. Groden; Irma Gutierrez; Sihoun Hahn; Rizwan Hamid; Neil A. Hanchard; Kelly Hassey; Nichole Hayes; Frances High; Anne Hing; Fuki M. Hisama; Ingrid A. Holm; Jason Hom; Martha Horike-Pyne; Alden Huang; Yong Huang; Laryssa Huryn; Rosario Isasi; Fariha Jamal; Gail P. Jarvik; Jeffrey Jarvik; Suman Jayadev; Lefkothea Karaviti; Emily G. Kelley; Jennifer Kennedy; Dana Kiley; Isaac S. Kohane; Jennefer N. Kohler; Deborah Krakow; Donna M. Krasnewich; Elijah Kravets; Susan Korrick; Mary Koziura; Joel B. Krier; Seema R. Lalani; Byron Lam; Christina Lam; Grace L. LaMoure; Brendan C. Lanpher; Ian R. Lanza; Lea Latham; Kimberly LeBlanc; Brendan H. Lee; Hane Lee; Roy Levitt; Richard A. Lewis; Sharyn A. Lincoln; Pengfei Liu; Xue; Zhong Liu; Nicola Longo; Sandra K. Loo; Joseph Loscalzo; Richard L. Maas; John MacDowall; Ellen F. Macnamara; Calum A. MacRae; Valerie V. Maduro; Marta M. Majcherska; Bryan C. Mak; May Christine V. Malicdan; Laura A. Mamounas; Teri A. Manolio; Rong Mao; Kenneth Maravilla; Thomas C. Markello; Ronit Marom; Gabor Marth; Beth A. Martin; Martin G. MartinJulian A. Martínez-Agosto; Shruti Marwaha; Jacob McCauley; Allyn McConkie-Rosell; Colleen E. McCormack; Alexa T. McCray; Elisabeth McGee; Heather Mefford; J. Lawrence Merritt; Matthew Might; Ghayda Mirzaa; Eva Morava; Paolo M. Moretti; Deborah Mosbrook-Davis; John J. Mulvihill; David R. Murdock; Anna Nagy; Mariko Nakano-Okuno; Avi Nath; Stan F. Nelson; John H. Newman; Sarah K. Nicholas; Deborah Nickerson; Shirley Nieves-Rodriguez; Donna Novacic; Devin Oglesbee; James P. Orengo; Laura Pace; Stephen Pak; J. Carl Pallais; Christina GS. Palmer; Jeanette C. Papp; Neil H. Parker; John A. Phillips III; Jennifer E. Posey; Lorraine Potocki; Bradley Power; Barbara N. Pusey; Aaron Quinlan; Wendy Raskind; Archana N. Raja; Deepak A. Rao; Genecee Renteria; Chloe M. Reuter; Lynette Rives; Amy K. Robertson; Lance H. Rodan; Jill A. Rosenfeld; Natalie Rosenwasser; Francis Rossignol; Maura Ruzhnikov; Ralph Sacco; Jacinda B. Sampson; Susan L. Samson; Mario Saporta; C. Ron Scott; Judy Schaechter; Timothy Schedl; Kelly Schoch; Daryl A. Scott; Vandana Shashi; Jimann Shin; Rebecca Signer; Edwin K. Silverman; Janet S. Sinsheimer; Kathy Sisco; Edward C. Smith; Kevin S. Smith; Emily Solem; Lilianna Solnica-Krezel; Ben Solomon; Rebecca C. Spillmann; Joan M. Stoler; Jennifer A. Sullivan; Kathleen Sullivan; Angela Sun; Shirley Sutton; David A. Sweetser; Virginia Sybert; Holly K. Tabor; Queenie K.-G. Tan; Mustafa Tekin; Fred Telischi; Willa Thorson; Audrey Thurm; Cynthia J. Tifft; Camilo Toro; Alyssa A. Tran; Brianna M. Tucker; Tiina K. Urv; Adeline Vanderver; Matt Velinder; Dave Viskochil; Tiphanie P. Vogel; Colleen E. Wahl; Stephanie Wallace; Nicole M. Walley; Chris A. Walsh; Melissa Walker; Jennifer Wambach; Jijun Wan; Lee-kai Wang; Michael F. Wangler; Patricia A. Ward; Daniel Wegner; Mark Wener; Tara Wenger; Katherine Wesseling Perry; Monte Westerfield; Matthew T. Wheeler; Jordan Whitlock; Lynne A. Wolfe; Jeremy D. Woods; Shinya Yamamoto; John Yang; Muhammad Yousef; Diane B. Zastrow; Wadih Zein; Chunli Zhao; Stephan Zuchner.

## Author Contributions

MJB, GPM, and LCM developed the approach for cell based killing assays and MPEG1 quantification. LCM conducted the experiments, analyzed and visualized data. KKP, JLB, and MJB provided clinical care. HL and SFN performed sequencing data analysis. SYJ organized data and wrote the first draft. All authors participated in manuscript editing. All authors have read and approved the submitted version.

## Funding

Perforin-2 research in the laboratory of GPM is supported by the National Institute of Allergy and Infectious Diseases of the National Institutes of Health under award number R01AI110810. MJB is supported by National Institutes of Health under R01 GM110482 and by the Jeffrey Modell Foundation. Sequencing and data analysis were supported by awards from the National Institutes of Health (NIH) Common Fund, U01HG007703 and the UCLA California Center for Rare Diseases of the Institute for Precision Health.

## Conflict of Interest

The authors declare that the research was conducted in the absence of any commercial or financial relationships that could be construed as a potential conflict of interest.

## References

[1] Mccormack RM Szymanski EP Hsu AP Perez E Olivier KN Goodhew EB MPEG1 / perforin-2 mutations in human pulmonary nontuberculous mycobacterial infections. JCI Insight (2017) 2:1–8. 10.1172/jci.insight.89635.Research PMC5396519 28422754

[2] McCormack RM de Armas LR Shiratsuchi M Fiorentino DG Olsson ML Lichtenheld MG Perforin-2 is essential for intracellular defense of parenchymal cells and phagocytes against pathogenic bacteria. Elife (2015) 4:1–29. 10.7554/eLife.06508 PMC4626811 26402460

[3] McCormack R Bahnan W Shrestha N Boucher J Barreto M Barrera CM Perforin-2 protects host cells and mice by restricting the vacuole to cytosol transitioning of a bacterial pathogen. Infect Immun (2016) 84:1083–91. 10.1128/IAI.01434-15 PMC4807494 26831467

[4] McCormack RM Lyapichev K Olsson ML Podack ER Munson GP Enteric pathogens deploy cell cycle inhibiting factors to block the bactericidal activity of Perforin-2. Elife (2015) 4:1–22. 10.7554/eLife.06505 PMC4626573 26418746

[5] Bai F McCormack RM Hower S Plano GV Lichtenheld MG Munson GP Perforin-2 Breaches the Envelope of Phagocytosed Bacteria Allowing Antimicrobial Effectors Access to Intracellular Targets. J Immunol (2018) 201:2710–20. 10.4049/jimmunol.1800365 PMC6200583 30249808

[6] Ni T Jiao F Yu X Aden S Ginger L Williams SI Structure and mechanism of bactericidal mammalian perforin-2, an ancient agent of innate immunity. Sci Adv (2020) 6:1–13. 10.1126/sciadv.aax8286 PMC6989145 32064340

[7] Karczewski KJ Francioli LC Tiao G Cummings BB Alföldi J Wang Q The mutational constraint spectrum quantified from variation in 141,456 humans. Nature (2020) 581:434–43. 10.1038/s41586-020-2308-7 PMC7334197 32461654

[8] Pang SS Bayly-Jones C Radjainia M Spicer BA Law RHP Hodel AW The cryo-EM structure of the acid activatable pore-forming immune effector Macrophage-expressed gene 1. Nat Commun (2019) 10:1–9. 10.1038/s41467-019-12279-2 31537793 PMC6753088

[9] Sherif RD Ingargiola M Sanati-Mehrizy P Torina PJ Harmaty MA Use of antibiotic beads to salvage infected breast implants. J Plast Reconstr Aesthet Surg (2017) 70:1386–90. 10.1016/j.bjps.2017.05.023 28651885

[10] De Ugarte DA Roberts RL Lerdluedeeporn P Stiehm ER Atkinson JB Treatment of chronic wounds by local delivery of granulocyte-macrophage colony-stimulating factor in patients with neutrophil dysfunction. Pediatr Surg Int (2002) 18:517–20. 10.1007/s00383-002-0733-3 12415398

[11] McCormack R De Armas LR Shiratsuchi M Ramos JE Podack ER Inhibition of intracellular bacterial replication in fibroblasts is dependent on the perforin-like protein (Perforin-2) encoded by macrophage-expressed gene 1. J Innate Immun (2013) 5:185–94. 10.1159/000345249 PMC3732477 23257510

[12] Strbo N Pastar I Romero L Chen V Vujanac M Sawaya AP Single cell analyses reveal specific distribution of anti-bacterial molecule Perforin-2 in human skin and its modulation by wounding and Staphylococcus aureus infection. Exp Dermatol (2019) 28:225–32. 10.1111/exd.13870 PMC7461719 30609079

[13] Pettersen EF Goddard TD Huang CC Couch GS Greenblatt DM Meng EC UCSF Chimera—a visualization system for exploratory research and analysis. J Comput Chem (2004) 25 13:1605–12. 10.1002/jcc.20084 15264254

